# Evaluation of Aqueous and Ethanolic Extracts for the Green Synthesis of Zinc Oxide Nanoparticles from *Tradescantia spathacea*

**DOI:** 10.3390/nano15141126

**Published:** 2025-07-20

**Authors:** Pedro Gerardo Trejo-Flores, Yazmin Sánchez-Roque, Heber Vilchis-Bravo, Yolanda del Carmen Pérez-Luna, Paulina Elizabeth Velázquez-Jiménez, Francisco Ramírez-González, Karen Magaly Soto Martínez, Pascual López de Paz, Sergio Saldaña-Trinidad, Roberto Berrones-Hernández

**Affiliations:** 1 Instituto de Investigación e Inovación en Energías Renovables, Universidad de Ciencias y Artes de Chiapas, Libramiento Norte Poniente No. 1150, Lajas Maciel, Tuxtla Gutiérrez 29039, Chiapas, Mexico; ptrejo@ip.upchiapas.edu.mx (P.G.T.-F.); heber.vilchis@unicach.mx (H.V.-B.); paulina.velazquez@unicach.mx (P.E.V.-J.); framirez@in.upchiapas.edu.mx (F.R.-G.); pascual.lopez@unicach.mx (P.L.d.P.); 2 Ingeniería en Nanotecnología, Universidad Politécnica de Chiapas, Carretera Tuxtla Gutiérrez-Portillo Zaragoza Km 21 + 500, Suchiapa 29150, Mexico, Mexico; yperez@upchiapas.edu.mx (Y.d.C.P.-L.); ssaldana@upchiapas.edu.mx (S.S.-T.); rberrones@upchiapas.edu.mx (R.B.-H.); 3 Centro de Investigación y de Estudios Avanzados del Instituto Politécnico Nacional, Instituto Politécnico Nacional, Libramiento Norte Poniente No. 2000, Real de Juriquilla, Santiago de Querétaro 76230, Querétaro, Mexico; karen.soto@cinvestav.mx

**Keywords:** aqueous extract, ethanolic extract, ZnO, pH, *Tradescantia spathacea*

## Abstract

In this work, we report a green synthesis of zinc oxide (ZnO) nanoparticles using aqueous and ethanolic extracts of *Tradescantia spathacea* (purple maguey) as bioreducing and stabilizing agents, which are plant extracts not previously employed for metal oxide nanoparticle synthesis. This method provides an efficient, eco-friendly, and reproducible route to obtain ZnO nanoparticles, while minimizing environmental impact compared to conventional chemical approaches. The extracts were prepared following a standardized protocol, and their phytochemical profiles, including total phenolics, flavonoids, and antioxidant capacity, were quantified via UV-Vis spectroscopy to confirm their reducing potential. ZnO nanoparticles were synthesized using zinc acetate dihydrate as a precursor, with variations in pH and precursor concentration in both aqueous and ethanolic media. UV-Vis spectroscopy confirmed nanoparticle formation, while X-ray diffraction (XRD) revealed a hexagonal wurtzite structure with preferential (101) orientation and lattice parameters a = b = 3.244 Å, c = 5.197 Å. Scanning electron microscopy (SEM) showed agglomerated morphologies, and Fourier transform infrared spectroscopy (FTIR) confirmed the presence of phytochemicals such as quercetin, kaempferol, saponins, and terpenes, along with Zn–O bonding, indicating surface functionalization. Zeta potential measurements showed improved dispersion under alkaline conditions, particularly with ethanolic extracts. This study presents a sustainable synthesis strategy with tunable parameters, highlighting the critical influence of precursor concentration and solvent environment on ZnO nanoparticle formation. Notably, aqueous extracts promote ZnO synthesis at low precursor concentrations, while alkaline conditions are essential when using ethanolic extracts. Compared to other green synthesis methods, this strategy offers control and reproducibility and employs a non-toxic, underexplored plant source rich in phytochemicals, potentially enhancing the crystallinity, surface functionality, and application potential of the resulting ZnO nanoparticles. These materials show promise for applications in photocatalysis, in antimicrobial coatings, in UV-blocking formulations, and as functional additives in optoelectronic and environmental remediation technologies.

## 1. Introduction

Nanotechnology has revolutionized multiple fields of research and industry by manipulating materials and structures at nanometer scales, leading to significant advances in fields as electronics, medicine, environmental remediation, and energy [1]. Nanoparticles (NPs), which are microscopic particles ranging in size from 1 to 100 nm, are fundamental components in these fields; due to their catalytic reactivity, chemical stability, thermal conductivity, and nonlinear optical properties derived from their surface-to-volume ratio [2].

Metal nanoparticles (MNPs) have recently attracted attention due to their unique physicochemical properties, low-cost production, and safety, as well as their multiple applications. These nanoparticles can be synthesized from different metals in addition to metal oxides such as Pt, Cu, Ag, Pd, NiO, CuO, Au, TiO_2_, and ZnO [3,4]. Several studies identify ZnO NPs as a remarkable semiconductor, with a wide and straight band gap (3.37 eV), temperature-dependent behavior, strong excitation binding energy (60 meV), and exceptional catalytic, antimicrobial, and antioxidant capabilities [5,6]. The application in photocatalysis, optical and electrical devices, cosmetics, antimicrobial coating, among others, are the extensive uses of zinc oxide nanoparticles (ZnO NPs) [7].

Chemical, physical, and biological methods are used for the synthesis of ZnO NPs; however, these technologies necessitate elevated temperature or pressure, hazardous chemicals, and high energy consumption, which generates a negative impact on the environment and public health [8,9]. Nevertheless, green synthesis, a biosynthetic process, has emerged as an ecological and economic alternative, taking advantage of biological extracts from plants, bacteria, fungi, or algae as reducing and stabilizing agents [10]. The utilization of plant extracts has gained popularity due to the presence of enzymes and secondary metabolites such as flavonoids, terpenoids, and phenols, which facilitate not only metallic ion reduction but also nanoparticle stabilization [11].

The morphological and structural characteristics of ZnO NPs synthesized via green methods are influenced by several factors, particularly the choice of extraction solvent, whether aqueous or ethanolic. The selected solvent affects the profile of metabolites, which subsequently influences the size, morphology, dispersion, and stability of the nanoparticles [12]. Research has shown that ethanolic extraction recovers a significant amount of phenolic and terpenoid compounds, while aqueous extraction is more effective for flavonoids and saponins. These differences influence the nucleation process and growth of nanoparticles [13]. Additionally, the pH value plays a crucial role in the reaction, as it affects the ionization of phytochemical functional groups, as well as crystallite size and morphology, ultimately influencing the nucleation of the nanoparticles [14,15].

Recent research has been published on the biosynthesis of ZnO NPs utilizing various natural plant extracts [13]. The Tradescantia genus is a promising source of bioactive compounds with stabilizing and reducing capabilities [16]. *Tradescantia spathacea*, commonly known as Maguey Morado, is a member of the Commelinaceae family and is native to regions in southern Mexico, Guatemala, and Belize. This plant has demonstrated biological properties, including antioxidant and anti-inflammatory effects, attributed to its content of alkaloids, coumarins, saponins, terpenoids, and flavonoids [17].

While other species such as *T. zebrina*, *T. fluminensis*, and *T. albiflora* have shown positive results in the biosynthesis of metallic nanoparticles, the application of *T. spathacea* in green synthesis remains limited [18]. Recent studies have reported its use in the production of SnO_2_ and Ni-SnO_2_ nanoparticles; however, there is a lack of research on its capability to synthesize metallic oxides, such as ZnO [19]. Therefore, this study focuses on the green synthesis of ZnO nanoparticles using aqueous and ethanolic extracts of *Tradescantia spathacea*, to evaluate how the phytochemical composition and pH of the synthesis medium influence the morphological and structural properties of the obtained nanoparticles.

## 2. Materials and Methods

In this work, *Tradescantia spathacea*, known in Mexico as purple maguey, is a plant used to extract phytochemicals that serve as a reducing agent to obtain ZnO nanoparticles. The phytochemicals, including flavonoids and phenolic compounds, as well as antioxidant activity, were obtained using the method described in this section.

### 2.1. Purple Maguey Leaves Preparation

The leaves were collected in the city of Tuxtla Gutiérrez, cleaned and disinfected using distilled water (DI) to remove impurities, and soaked for 10 min in a solution of 10% ethanol (Meyer, 96%,Ciudad de México, Mexico) and DI. Subsequently, the leaves were cut into ~2 × 2 cm and separated into two groups. The group labeled as fresh was where the leaves were stored at 8 °C in airtight bags without other treatment. On the other hand, the group labeled as dry was where the leaves were dried at 60 °C for 7 days and then stored in airtight bags.

### 2.2. Method to Obtain the Aqueous Extract

Two aqueous extracts were obtained from the fresh and dry groups, one from each one. An amount of mass (g) of the leaves was mixed in the DI for 20 min at 60 °C, under stirring (250 rpm). The aqueous extracts were (1) 100 g of fresh leaves with 400 mL DI and (2) 30 g dry leaves with 300 mL DI. Finally, both aqueous extracts were filtered through Whatman No. 1 and stored in amber bottles at 4 °C [20].

### 2.3. Method to Obtain the Ethanolic Extract

Two ethanolic extracts were obtained from the dry group. The amount of mass of the leaves was 30 g mixed in ethanol for 20 min at 60 °C, under stirring (250 rpm). The amount of ethanol was (1) 200 mL and (2) 500 mL. Finally, both aqueous extracts were filtered through Whatman No. 1 and stored in amber bottles at 4 °C [21].

### 2.4. Determination of Phenolic and Antioxidant Compounds in the Extracts

The techniques used to measure phytochemical compounds in aqueous and ethanolic extracts are described below. Therefore, the term “extract” is used since the methodology used was the same for both. The determinations were performed in triplicate [22].

#### 2.4.1. Total Phenol Content

The spectrophotometric method described in [22] was used, in which 0.125 mL of extract was added, mixed with 0.625 mL of the Folin–Ciocalteu (Golden bell, 2N, Ciudad de México, Mexico) reagent diluted in deionized water (1:10) and 0.5 mL of 7.5% Na_2_CO_3_ solution. After 45 min of standing, the absorbance at 760 nm was measured at room temperature without light. The total phenol content was calculated by a calibration curve using Gallic acid (Meyer, 97%, Ciudad de México, Mexico) as a standard. The results were expressed as mg eq. of Gallic acid/g of extract.

#### 2.4.2. Total Flavonoid Content

It was determined under the formation of an aluminum–flavonoid complex. To 250 μL of extract, 1.25 mL of distilled water and 75 μL of NaNO_2_ (Meyer, 97%, Ciudad de México, Mexico) (5%) were added; after 5 min of standing, 150 μL of AlCl_3_ (Meyer, 97%, Ciudad de México, Mexico) (10%) were added. Subsequently, 500 μL of NaOH (Meyer, 97%, Ciudad de México, Mexico) (1 M) and 275 μL of distilled water were added, the sample was shaken vigorously, and quantification was performed in a UV-Vis spectrophotometer at 510 nm. The standard was prepared with quercetin (Thermo Scientific Chemicals, 95%, Waltham, MA, USA) dissolved in absolute ethanol, which allowed for obtaining the calibration curve. The results were expressed in mg/g Eq. of quercetin [23].

#### 2.4.3. Evaluation of Antioxidant Capacity

The antioxidant capacity was determined following the methodology described in [24] with some modifications. A total of 200 μL of extract was added to 2 mL of DPPH (Sigma-Aldrich, level 100, St. Louis, MI, USA) solution (125 μM in 100 mL of 80% methanol (Meyer, 99.8%, Ciudad de México, Mexico) and left to stand for 60 min in the absence of light. The absorbance of the solution was measured at 520 nm. A calibration curve with TROLOX (Sigma-Aldrich, 98%, St. Louis, MI, USA) as a standard was used. The results were expressed in mg/g Eq of TROLOX.

### 2.5. Synthesis of ZnO Nanoparticles

Zinc acetate dihydrate (Fermont, 99.3%, Monterrey, NL, México) was used as zinc oxide precursor. The precursor was diluted in distilled water (DI) in concentrations of 0.1 M and 0.5 M to obtain precursor solutions. The ZnO nanoparticles were synthesized by mixing 40 mL of each precursor solution with 20 mL of each extract, aqueous and ethanolic. The mixtures were under stirring (250 rpm) for 2 h, at 60 °C, and allowed to cool to room temperature. The next step was the adjustment of the pH, using NaOH (0.5 M), to 7 and 10 for each mixture, which resulted in a precipitate. The precipitated material was centrifuged at 3500 rpm for 30 min, and the sediment particles were recovered and washed twice with DI, followed by two washes with ethanol. The particles were dried at 75 °C for 8 h and calcined at 400 °C for 2 h, obtaining a grayish powder, which was stored in capped tubes at room temperature [25,26]. The resulting particles were labeled with letters and numbers XY00/00 (X = extract medium: aqueous or ethanolic; Y = leaves group: fresh or dry; 00 = precursor concentration: 0.5 M or 0.1 M; pH: 7 or 10). Table 1 summarizes the experimental conditions used to synthesize ZnO NPs.

### 2.6. Characterization of ZnO NPs

The content of phenols, flavonoids, and antioxidant capacity, from the aqueous and ethanolic extracts, was determined by spectrophotometric measurements, using a UV-Vis (LOVIBOND XD 7500, (Lovibond, Dortmund, Alemania) wavelength range 190–1100 nm).

Regarding the ZnO NPs, the morphology was obtained using Field Emission Scanning Electron Microscopy (FE-SEM) with a JEOL Ltd equipment model JSM-7100F (Akishima, Tokio, Japan), operating at 15 kV and with a resolution of 2 nm. The chemical composition was determined by Fourier transform infrared (FTIR) spectroscopy, within the wavenumber range from 500 to 4000 cm^−1^. The measurements were carried out with a PerkinElmer spectrometer, model Spectrum Two (Waltham, MA, USA), in diffuse reflectance mode, with 24 scans and a resolution of 4 cm^−1^. The samples were characterized in powder, which was pressed into a potassium bromide holder. The crystal structure was analyzed by X-ray diffraction (XRD) patterns obtained with a Rigaku diffractometer, model ULTIMA IV (Akishima, Tokio, Japan), operating at 44 kV and 20 mA wth λ = 1.541 Å. The data were collected using the Bragg–Brentano geometry (2θ) from 5° to 80° with 0.02° steps. The XRD patterns were indexed using the Rigaku X-ray Powder Diffraction Software PDXL ver. 1.8.0.3. The Debye–Scherrer equation was used to calculate the crystallite size with K = 0.9 [27,28,29].

The hydrodynamic size and zeta potential were measured by Dynamic Light Scattering (DLS) with Anton Paar Litesizer 500 equipment, Kalliop ver. 1.2.0 (serial number 82124106, Australia), with a resolution range from 0.3 nm to 10 µm. In this characterization, the ZnO particles were dispersed by sonication in distilled water. The optical properties of the NPs were evaluated using UV-Vis spectrophotometry (SpectraMax Tunable Microplate Reader, Molecular Devices Co., Sunnyvale, CA, USA) across wavelengths from 200 to 1200 nm [28].

## 3. Results and Discussion

### 3.1. UV-Vis Characterization of the Purple Maguey Extracts

The content of phenols, flavonoids, and antioxidant activity of the aqueous and ethanolic extracts, obtained from purple maguey (*Tradescantia spathacea*), was determined by UV-Vis. The results are shown in Table 2, where the presence of the measured phytochemical content is presented. It is observed that the aqueous dry extract has a slightly higher content of phenols, flavonoids, and antioxidants than the aqueous fresh one; in contrast, the phytochemical concentration is higher in the ethanolic dry 1 extract, due to the difference in the amount of ethanol used during the extraction.

It has been reported that the phytochemicals carry out the green synthesis of nanoparticles, since this technique is based on the reduction of metals by natural species with antioxidant power, which are extracted from plants [30].

In green synthesis, several authors indicate that the number of phytochemical compounds present in the extracts is not a determining variable in the efficiency during the synthesis of nanoparticles, but the main factor responsible for the formation of nanoparticles is the set of flavonoid and phenol compounds that are present [31,32,33,34,35,36]. The set of compounds is inherent to the plant and depends on the growing conditions. The variation on the set of compounds may be due to intrinsic factors such as age, parts, and family of the plant and extrinsic factors such as climate, soil, water, light, stress, etc. Likewise, the result of extraction may be related to the chemical composition of the phenolic compounds and the used solvent, since they have a high solubility in water, making an aqueous extraction efficient, while the use of solvents is directed to lipophilic compounds [33]. On these variation basis, the results in Table 2 differ compared to those reported by Tan et al. [31], who made an ethanolic extract, reporting a phenol content of 203.9 ± 16.3 (mg Eq. Gallic acid/100 g of sample) and flavonoids of 10.8 ± 2.9 (mg Eq. quercetin/100 g of sample); on the other hand, Rosales-Reyes et al. [32] made an aqueous extract and reported a phenol content of 2100 (mg Eq. Gallic acid/L) for fresh leaves and 3010 (mg Eq. Gallic acid/L) for dry leaves; as for antioxidant activity, they reported 26.3 (-D.O. −3/min/mgm.s) for fresh leaves and 29.9 (-D.O. −3/min/mgm.s) for dry leaves.

### 3.2. Characterizations of ZnO NPs Obtained by Green Synthesis

Different experiments of green synthesis of ZnO NPs, using aqueous and ethanolic extracts, were implemented according to Table 1. In all cases, a white powder was obtained, which was subjected to different characterizations [35].

#### 3.2.1. UV-Vis Spectrophotometry

**Figure 1:**
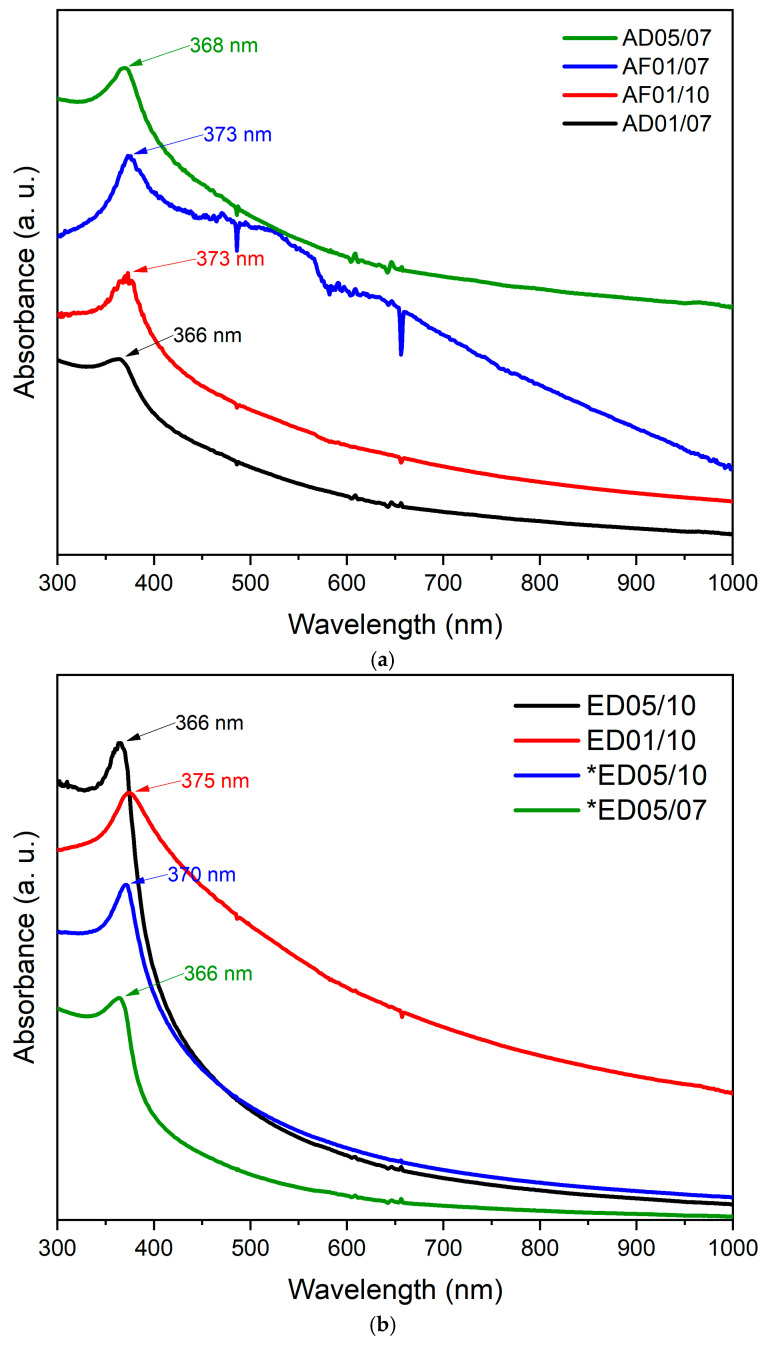
(**a**) UV-Vis spectra of ZnO NPs synthesized with phytochemicals obtained from the aqueous extracts: AD01/07, AF01/10, AF01/07, and AD05/07. (**b**) UV-Vis spectra of ZnO NPs synthesized with phytochemicals obtained from the ethanolic extracts: ED05/10, ED01/10, *ED05/10, and *ED05/07. The asterisk (*) stands for the ethanolic extracts obtained using 500 mL of ethanol.

UV-Vis spectra of ZnO NPs synthesized with aqueous and ethanolic extracts are shown in Figure 1a,b, respectively. The spectra have an average peak of ~368 nm, typical of zinc oxide, and are reported as an indicator of a successful synthesis; the bad range has been reported to be 360 to 400 nm [37]. The spectra of the ZnO NPs (Figure 1a) from samples AF01/10, AF01/07, and AD05/07 presented a sharper shape than AD01/07, which was made using a pH of 7. On the other hand, AF01/10 has the sharpest peak among the others (AF01/10 and AF01/07), which was made using a pH of 10. Some authors reported that the shape of the peak is related to the mono or polydispersity regarding the sharper or wider profile, respectively [38].

Figure 1b shows the results of the ethanolic samples. The samples made with ethanolic extract using 200 mL of ethanol, ED05/10 and ED01/10 (same pH), present a significant difference in the peak profile; sample ED05/10 presents a sharper absorbance peak, which was made using 0.5 M. In this case, it is observed that, when the pH is the same, the molar concentration affects the peak profile. On the other hand, the absorbance in samples ED05/10 and ED05/07 contain the same volume of solvent (500 mL of ethanol) but different pH, and it is observed that ED05/07 (pH 7) has the narrowest peak, confirming that higher pH produces ZnO NPs with a sharper absorbance peak profile by UV-vis at ~368 nm. It should be noted that, in results in which the bands observed are wider and less pronounced, the ZnO NPs approach to polydispersity (nanoparticles of different sizes) [38,39,40,41,42,43,44,45].

UV-vis was used as an initial method to evaluate the synthesis, but not all of the samples presented the absorbance peak (~365 nm); therefore, these were not considered for the following characterizations, and the crystallite size in Table 3 is blank. The results of UV-vis indicate that, to synthesize ZnO using aqueous extracts, low concentrations of precursor (0.1 M) in an alkaline environment are necessary, because higher concentrations do not produce ZnO due to the saturation of the solution; this is the case for samples AF05/07, AF05/10, and AD05/10. The balance between the precursor concentration and the synthesis environment is a key parameter for obtaining ZnO. When using ethanolic extracts, the pH is crucial regardless of concentration. This is the case for samples ED05/07, ED01/07, and *ED01/07, whose pH was 7.

#### 3.2.2. XRD Characterization

**Figure 2:**
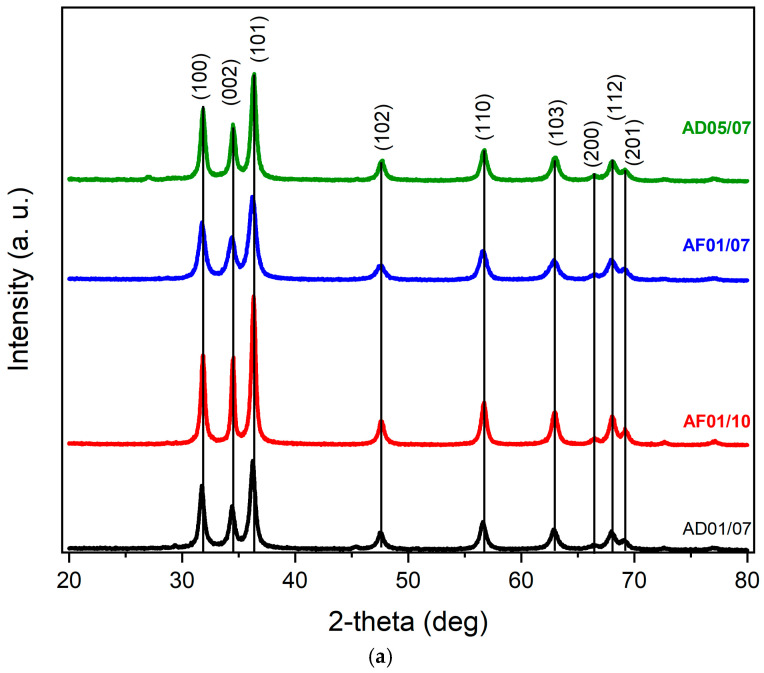

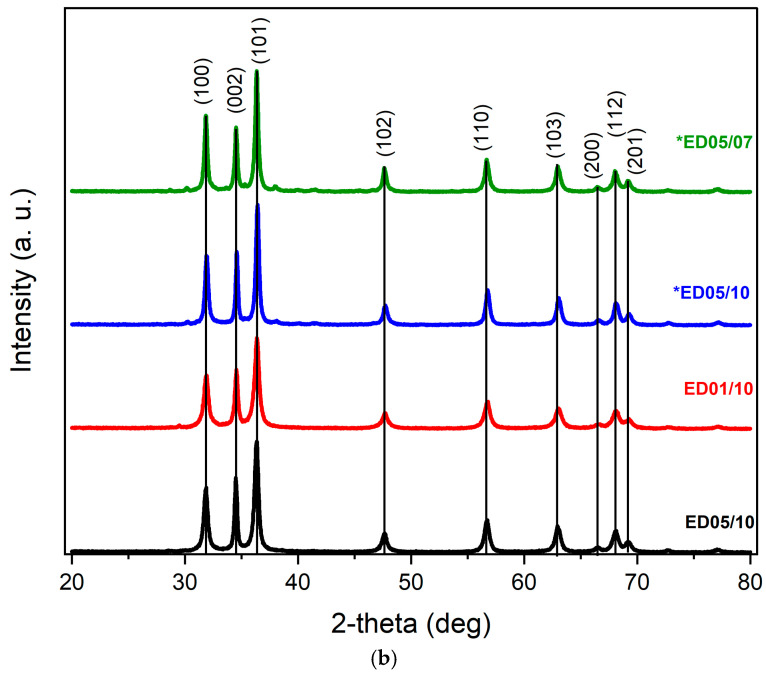
(**a**) XRD of ZnO NPs synthesized using phytochemicals obtained from the aqueous extracts: AD01/07, AF01/10, AF01/07, and AD05/07. (**b**) XRD of ZnO NPs synthesized using phytochemicals obtained from the ethanolic extracts: ED05/10, ED01/10, *ED05/10, and *ED05/07. The asterisk (*) stands for the ethanolic extracts obtained using 500 mL of ethanol.

XRD patterns of ZnO NPs, synthesized using the aqueous and ethanolic extracts, are shown in Figure 2a,b, respectively. The results indicate that all of the measured samples have the hexagonal wurtzite phase of ZnO, indexed with PDF 01-070-8072, which has the crystallographic planes (100), (002), (101), (102), (110), (103), (200), (112), and (201), at 31.84°, 34.50°, 36.34°, 47.65°, 56.73°, 63.01°, 66.54°, 68.12°, and 69.26°, respectively. All the samples have the preferential growth plane (101), and the lattice parameters a, b, and c were calculated using Equations (1) and (2) and the measured angle of diffraction of the planes (100) and (002), respectively [46]; the calculated parameters, assuming a=b due the hexagonal wurtzite structure, are summarized in Table 3.

(1) $$a_{\left(100\right)}=\frac{\lambda }{\sqrt{3}\sin\theta }$$

(2) $$c_{\left(002\right)}=\frac{\lambda }{\sin\theta }$$

The interplanar space (d) was calculated using Bragg’s Equation, nλ=2dsinθ, with λ=1.541 Å and n=1. According to PDF 01-070-8072, used to index the diffraction peaks, the interplanar spacings for the planes (100) and (002) are 2.812 Å and 2.602 Å, respectively. It is observed that the PDF and the calculated values are similar; therefore, we assume that the ZnO NPs are crystalline [47,48].

For crystal size analysis, it must be considered that macroscopic samples of crystalline materials, such as metals, semiconductors, and insulators, are not necessarily composed of a single crystal but rather are formed by a set or conglomerate of small crystals, which may have different orientations, separated from each other by boundaries or grain limits [49,50].

Crystal size refers to the measurement (size) of a single crystal in a polycrystalline material. In materials with a high degree of crystallinity, such as metals, where the material is composed of small crystals (crystallites) that are fused together, the crystallite size has an impact on the mechanical properties of the material. Thus, a reduction in crystal size causes diffraction peaks, when the size of a crystallite is smaller than 0.1 µm (1000 Å), to broaden. If the size of the crystallite is assumed to be the size of a coherently diffracting domain, then the particle size is not the crystallite size [50,51,52,53].

The Debye–Scherrer Equation, $D=\frac{0.9\lambda }{\beta cos\theta }$, was used to calculate the crystallite size; D is the crystallite size, λ is the X-ray wavelength (1.541 Å), β is the peak width half-width (FWHM), and θ is the diffraction angle, with the last two expressed in radians. The diffraction peaks regarding crystallographic planes (100), (002), and (101) were used to calculate the crystallite sizes due to their relation to the lattice constants and the preferential growth plane; the sizes are shown in Table 3, which are similar to those reported in [47,48,49]. It is observed that the AF01/10 (pH 10) sample presents the largest crystallite size among the aqueous samples. The samples synthesized using the same pH (AD01/10, AF01/07, and AD05/07) have similar crystalline size. Then, the pH affects the crystalline size; it is reported that larger crystallite sizes are generally associated with monodisperse systems, where crystallites have a more uniform size distribution [54].

Samples ED05/10 and ED010, made with ethanolic extract using a pH of 10 and 200 mL of ethanol, present similar crystallite sizes, 25 nm and 22 nm; the crystallite is slightly larger in ED05/10, and it is proposed that the difference is due to the higher concentration of 0.5 M. On the other hand, samples ED05/10 and ED05/07 contain the same volume of solvent (500 mL of ethanol) but different pH. It is observed that the crystallite sizes are close but slightly smaller in ED05/07 (pH 7), and we assume that the higher precursor concentration increases the nucleation [55].

#### 3.2.3. SEM Analysis

**Figure 3:**
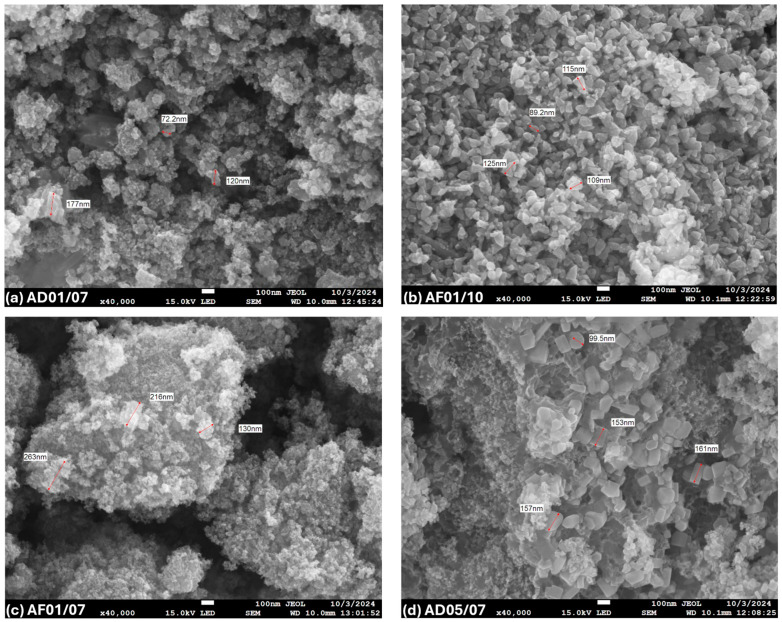
SEM of ZnO NPs synthesized using phytochemicals obtained from the aqueous extracts: (**a**) AD01/07, (**b**) AF01/10, (**c**) AF01/07, and (**d**) AD05/07. This evaluation represents the average of three repetitions.

The morphology of the nanoparticles obtained using both extracts was determined by SEM analysis. Figure 3 shows the images of the aqueous extracts in which it can be observed that (a)-AD01/07 presented an irregular morphology (Figure 3a); likewise, in (c)-AF01/07, spherical nanoparticles with a marked agglomeration are observed, indicating that neutral pH and 0.1 M concentration favor the growth of polydisperse particles tending to agglomerate [5]. Sample (d)-AD05/07, synthesized with a higher concentration (0.5 M) and pH of 7, generated nanoparticles with prismatic or hexagonal morphology with moderate agglomeration.

Finally, (b)-AF01/10, synthesized at a pH of 10 and 0.1 M, showed uniform pseudospherical particles, with low agglomeration and homogeneous distribution tending to monodisperse, standing out as the optimal sample, since it exhibits a uniform shape, highlighting the influence of a pH of 10 to generate these morphologies; this result is consistent with UV-vis and DRX measurements.

**Figure 4:**
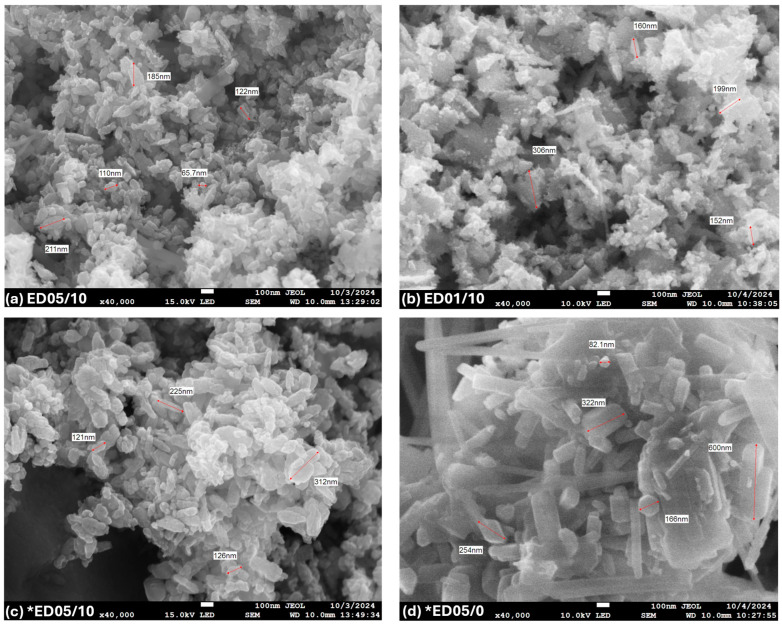
SEM of ZnO NPs synthesized using phytochemicals obtained from the ethanolic extracts: (**a**) ED05/10, (**b**) ED01/10, (**c**) *ED05/10, and (**d**) *ED05/07. The asterisk (*) stands for the ethanolic extracts obtained using 500 mL of ethanol. This evaluation represents the average of three repetitions.

Figure 4 shows the images corresponding to the ethanolic extracts, both with pH 10 and 200 mL of ethanol but with different precursor concentrations. In sample ED05/10 (0.5 M, Figure 4a), more uniform nanoparticles are observed, with a hemispherical and compact morphology, unlike ED01/10 (0.1 M, Figure 4b), which showed less homogeneous particles, an effect attributable to the lower concentration of precursors, which reduces the nucleation density [55]. On the other hand, when the precursor concentration is the same but the pH varies, as in samples (c)-*ED05/10 (d)-*ED05/07, the neutral pH tends to promote bastons among particles that are polydisperse (Figure 4d). Finally, the morphology, shown in Figure 4c, of the particles tends to uniformity in sample (c)-*ED05/10, which is favored by more alkaline conditions [56,57].

In both extracts, samples AF01/10 and ED05/10 (same pH) exhibited improved properties, including greater uniformity, low agglomeration, homogeneous distribution, and a hemispherical shape.

Nanoparticle size measurements, shown in Figure 3 and Figure 4, were performed manually from SEM micrographs with a resolution of 1280 × 1024 pixels, acquired at 40,000× magnification, 15 kV accelerating voltage, and a scale bar of 100 nm. The average relative error was estimated to be approximately 6.25%, based on a manual measurement uncertainty of ±5 nm. This uncertainty was determined by considering the pixel size at the specified magnification, the image resolution, and the precision limitations of the analysis software (PC SEM, provided by JEOL).

#### 3.2.4. FTIR Analysis

**Figure 5:**
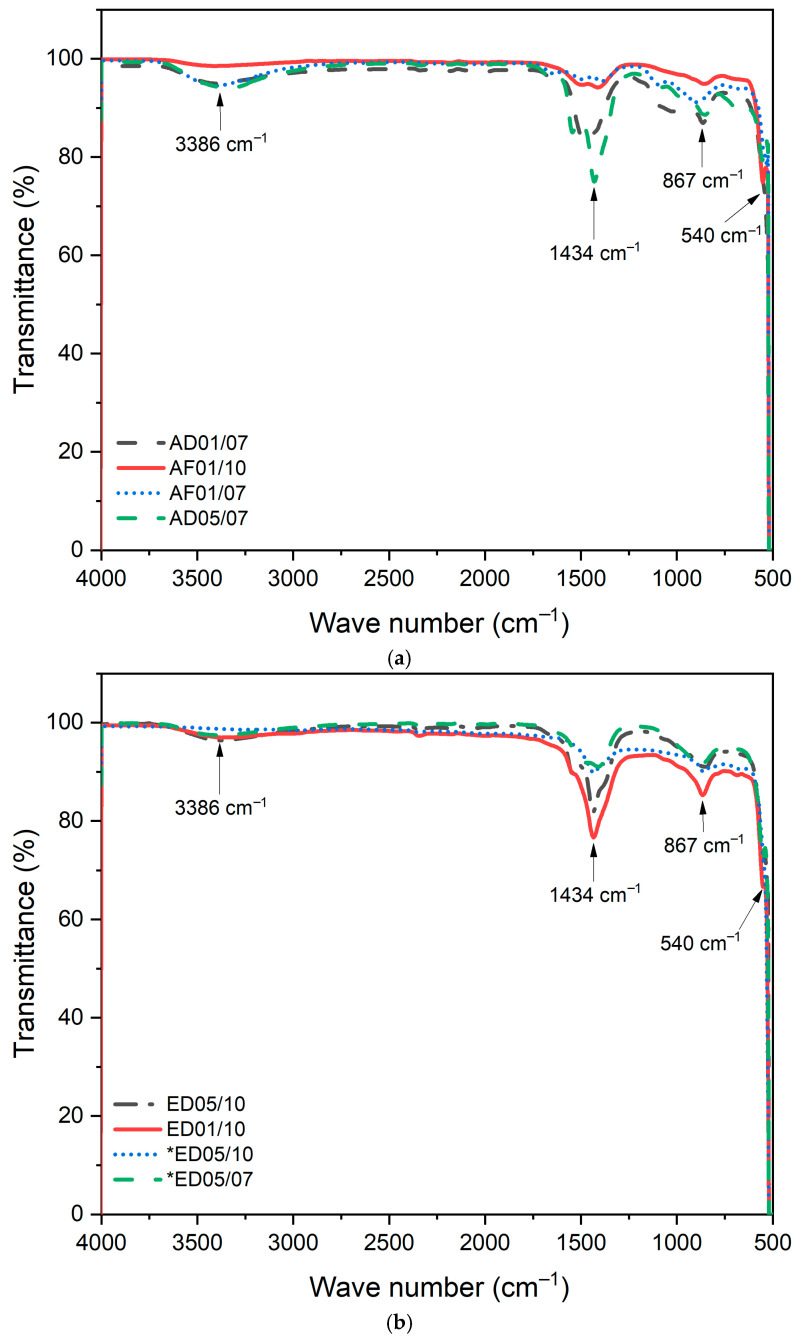
(**a**) FTIR of ZnO NPs synthesized using phytochemicals obtained from the aqueous extracts: AD01/07, AF01/10, AF01/07, and AD05/07. (**b**) FTIR of ZnO NPs synthesized using phytochemicals obtained from the ethanolic extracts: ED05/10, ED01/10, *ED05/10, and *ED05/07. The asterisk (*) stands for the ethanolic extracts obtained using 500 mL of ethanol.

Fourier transform infrared spectroscopy (FTIR) was used to identify the functional groups in the samples, and the results are shown in Figure 5a,b, for aqueous and ethanolic extracts. In both cases, the bands located between 3390 and 3385 cm^−1^ are related to the elongation vibrations of the -OH group, indicating the presence of alcohols and phenols. The band in the range of 1545 to 1411 cm^−1^ corresponds to elongation vibrations of C=C or C=O bonds [58], which are linked to kaempferol, due to their aromatic rings, and to phenolic acids. Furthermore, the band observed in the range of 894 to 856 cm^−1^ is related to the vibrations of the C-H bond, typical of some organic compounds such as flavonoids and terpenes (limonene and pinene), which are present in smaller quantities in the purple maguey. Finally, the band around 400–600 cm^−1^ affirms the vibrations of the Zn-O bond, confirming the formation of zinc oxide nanoparticles [59]. The synthesis of ZnO nanoparticles is possibly due to the interaction of phenolic compounds, alkaloids, terpenoids, and flavonoids from the purple maguey; these results suggest that the functional groups are responsible for reducing Zn^+^ ions to ZnO [60].

It should be noted that the extraction method used did not have a significant effect on the identification of the characteristic bands, since the same composition was observed in both cases. However, ethanolic extraction allowed for better definition of the bands for analysis [60,61,62].

In the aqueous extracts, the AF01/10 treatment (pH 10, 0.1 M) presented the most defined bands, particularly in the regions around 1434 cm^−1^ (carboxylate vibrations) and 540 cm^−1^ (Zn–O), indicating a more efficient surface functionalization, favored by the deprotonation of –OH and –COOH groups at alkaline pH, promoting their coordination with ZnO. In contrast, AD01/07 (pH 7, 0.1 M) has broader and less intense bands, reflecting a weak interaction between the polar metabolites of the extract, attributable to the protonation of the functional groups and the low concentration of the precursor [20,26,29]. In the ethanolic extracts, ED01/10 (pH 10, 0.1 M) showed the most intense and sharp bands in the characteristic regions of Zn–O, C=O, and O–H, indicating a strong organic interaction favored by less polar compounds with a higher affinity for the ZnO surface. ED05/07 (pH 7, 0.5 M) presented soft and diffuse bands, indicating less efficient surface functionalization [45,56,61].

#### 3.2.5. DLS and Z Potential Analysis

The average particle size in AF01/10 is 95 nm; these particles were synthesized in an alkaline ambient (pH 10), among the aqueous extracts. Meanwhile, *ED05/10, synthesized using 500 mL of ethanol and pH 10, has an average particle size of 61 nm. It is observed that the pH has an important role in the ZnO NPs synthesis [63,64,65,66]; these results agree with UV-vis and XRD.

**Figure 6:**
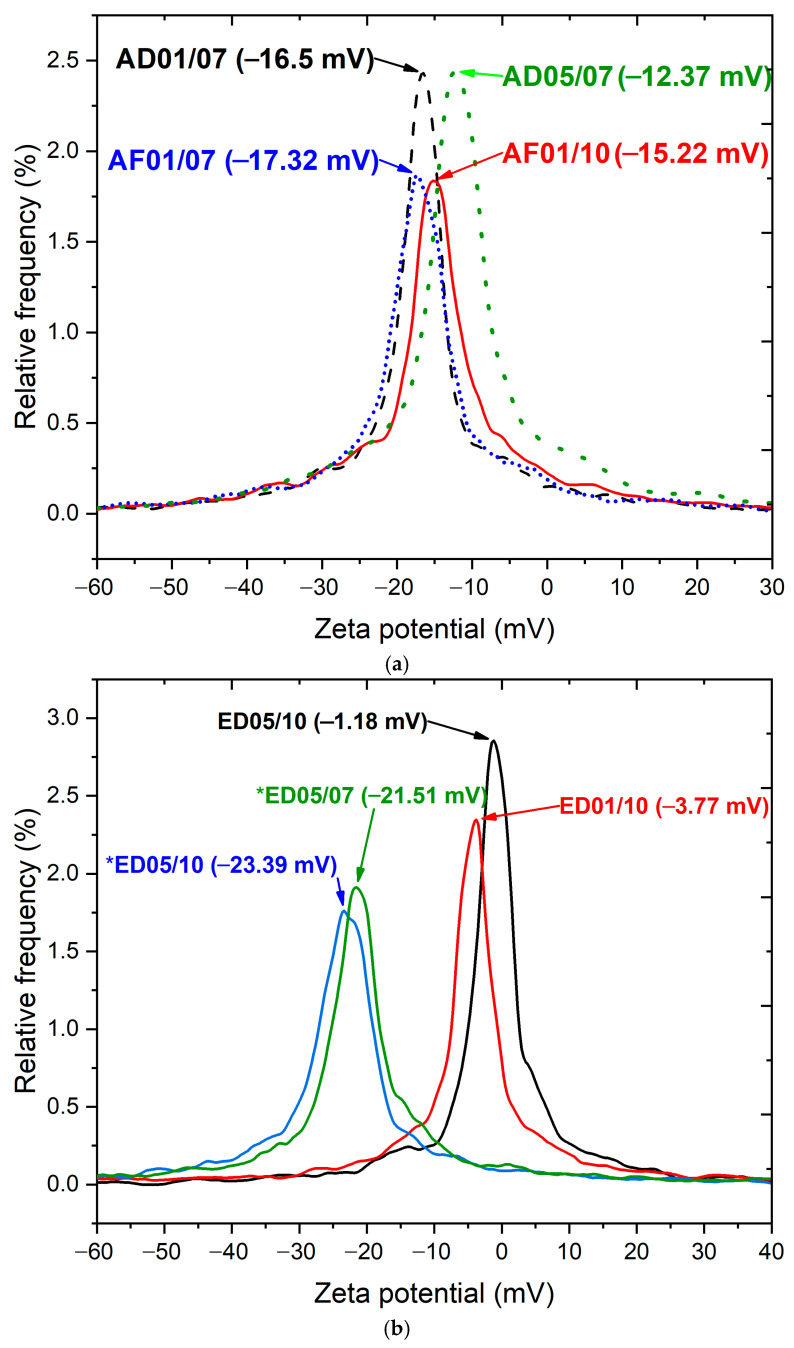
(**a**) Intensity of the Z potential of ZnO NPs synthesized using phytochemicals obtained from aqueous extracts: AD01/07, AF01/10, AF01/07, and AD05/07. (**b**) Intensity of the Z potential of ZnO NPs synthesized using phytochemicals obtained from ethanolic extracts: ED05/10, ED01/10, *ED05/10, and *ED05/07. The asterisk (*) stands for the ethanolic extracts obtained using 500 mL of ethanol.

Z potential values are important because they allow for determining the colloidal stability of the schematized particles and their potential applications, such as in water bio-remediation and in biological systems, such as lipid enhancement in microalgae [67,68,69,70,71,72]. The Z potential was determined for both extracts, and the results are shown in Figure 6a,b. In the aqueous samples, a value greater than ±10 mV was observed (Figure 6a), indicating that the particles exhibited greater colloidal stability, generating greater dispersion in a liquid medium. In the ethanolic extracts (Figure 6b), the result was close to 0 mV in the samples with a low ethanol concentration (200 mL, ED05/10, ED01/10), making the nanoparticles unstable and more prone to aggregation. Contrarily, a greater ethanol concentration (500 mL, ED05/10, *ED05/07) promotes greater colloidal stability. This result allows us to determine the starting experimental conditions to synthesize ZnO particles with targeted colloidal stabilization characteristics [72,73,74].

## 4. Conclusions

In this work, we demonstrated that aqueous and ethanolic extracts of *Tradescantia spathacea* (commonly known as purple maguey) can act as effective reducing and stabilizing agents for the green synthesis of ZnO nanoparticles. The extracts exhibited distinct phytochemical profiles, particularly in phenolic and flavonoid content, depending on the solvent type, leaf condition (fresh or dried), and extract volume. Notably, aqueous extracts from dried leaves contained higher concentrations of antioxidant compounds, while the phytochemical content of ethanolic extracts varied with ethanol concentration and extraction parameters.

ZnO nanoparticles were synthesized using zinc acetate dihydrate as a precursor under varying pH and precursor concentration conditions. UV-Vis analysis confirmed nanoparticle formation in both aqueous and ethanolic systems. Structural and morphological characterizations revealed that pH had a more significant impact than precursor concentration on nanoparticle crystallinity, morphology, crystallite size, hydrodynamic diameter, and colloidal stability. Aqueous extracts enabled ZnO formation at low precursor concentrations, whereas alkaline conditions were essential when using ethanolic extracts.

The novelty of our approach lies in (i) the systematic evaluation of both aqueous and ethanolic extracts of *T. spathacea*, an underexplored plant species, and (ii) the clear identification of synthesis parameters, particularly pH and precursor concentration, that govern nanoparticle quality and stability. Compared to other green synthesis methods that often lack precise control over these factors or do not fully characterize the extract composition [75,76], our method provides a reproducible and tunable protocol for the production of ZnO nanoparticles with enhanced colloidal behavior and surface functionalization.

These findings establish a foundation for scalable, eco-friendly ZnO nanoparticle production with potential applications in antimicrobial coatings, UV-blocking formulations, and photocatalysis. Future work will focus on assessing the bioactivity of these nanoparticles and tailoring their properties for specific functional applications.

Finally, we propose that ZnO nanoparticles synthesized under ED05/10 conditions (ethanolic extract with optimized pH and precursor concentration) are particularly suitable for dispersion-based applications, such as enhancing lipid accumulation in microalgae cultures. Further investigation into their biological interactions and catalytic performance will support the development of advanced biogenic nanomaterials for sustainable technologies.

## Figures and Tables

**Table 1:** Experimental conditions to synthesize ZnO NPs by green synthesis.

Sample Label	Extract Type	Leaves Group	Precursor Concentration (M)	pH
AD01/07	Aqueous	Dry	0.1	7
AF01/10	Aqueous	Fresh	0.1	10
AF01/07	Aqueous	Fresh	0.1	7
AD05/07	Aqueous	Dry	0.5	7
AF05/07	Aqueous	Fresh	0.5	7
AD01/10	Aqueous	Dry	0.1	10
AF05/10	Aqueous	Fresh	0.5	10
AD05/10	Aqueous	Dry	0.5	10
ED05/10	Ethanolic	Dry	0.5	10
ED01/10	Ethanolic	Dry	0.1	10
* ED05/10	Ethanolic	Dry	0.5	10
* ED05/07	Ethanolic	Dry	0.5	7
ED05/07	Ethanolic	Dry	0.5	7
ED01/07	Ethanolic	Dry	0.1	7
* ED01/07	Ethanolic	Dry	0.1	7
* ED01/10	Ethanolic	Dry	0.1	10

* These ethanolic extracts were made with 500 mL of ethanol.

**Table 2:** Content of phenols, flavonoids, and antioxidant capacity, from the aqueous and ethanolic extracts, obtained by spectrophotometric measurements.

Extract	Phenols (mg Eq.) Gallic Acid/g	Flavonoids (mg Eq.) Quercetin/g	Antioxidant (mg Eq.) TROLOX/g
Aqueous Fresh	759.55 ± 33.2	16.50 ± 1.5	18.39 ± 4.5
Aqueous Dry	1152.96 ± 8.9	21.02 ± 11.4	33.54 ± 9.0
Ethanolic Dry 1 *	829.50 ± 12.8	23.42 ± 2.2	39.60 ± 13.6
Ethanolic Dry 2 *	777.24 ± 52.2	12.03 ± 3.0	21.42 ± 4.5

* The ethanolic extracts were obtained using (1) 200 mL and 500 mL (2) of ethanol.

**Table 3:** Calculated lattice parameters.

Sample	$2\theta_{\left(100\right)}$ (deg)	FWHM (deg)	$d_{\left(100\right)}$ (Å)	$2\theta_{\left(002\right)}$ (deg)	FWHM (deg)	$d_{\left(002\right)}$ (Å)	a=b (nm)	c (nm)	Crystallite Size (nm)	Particle Size (nm)
AD01/07	31.828	0.397	2.808	34.493	0.297	2.597	3.244	5.197	18	424
AF01/10	31.831	0.210	2.807	34.487	0.145	2.597	3.244	5.198	23	95
AF01/07	31.799	0.598	2.810	34.393	0.495	2.604	3.247	5.212	11	280
AD05/07	31.753	0.467	2.814	34.340	0.409	2.608	3.252	5.220	17	430
ED05/10	31.821	0.400	2.808	34.533	0.248	2.594	3.245	5.191	25	126
ED01/10	31.780	0.300	2.812	34.501	0.290	2.596	3.249	5.196	22	541
* ED05/10	31.906	0.316	2.801	34.554	0.182	2.592	3.237	5.188	32	61
* ED05/07	31.845	0.278	2.806	34.544	0.229	2.593	3.243	5.190	31	499

The asterisk (*) stands for the ethanolic extracts obtained using 500 mL of ethanol.

## Data Availability

All data that support the findings of this study are included within the article.

## References

[1] Castañeda-Naranjo L.A., Palacios-Neri J. (2014). Nanotechnology: Source of new paradigms. Mundo Nano Rev. Interdiscip. Nanocienc. Nanotecnol..

[2] Ijaz I., Gilani E., Nazir A., Bukhari A. (2020). Detail review on chemical, physical and green synthesis, classification, characteriza-tions and applications of nanoparticles. Green Chem. Lett. Rev..

[3] Nasrollahzadeh M., Sajjadi M., Sajadi S.M., Issaabadi Z. (2019). Green Nanotechnology. Interface Sci. Technol..

[4] Saleh T.A. (2020). Nanomaterials: Classification, properties, and environmental toxicities. Environ. Technol. Innov..

[5] Sirelkhatim A., Mahmud S., Seeni A., Kaus N.H.M., Ann L.C., Bakhori S.K.M. (2015). Review on Zinc Oxide Nanoparticles: Antibacterial Activity and Toxicity Mechanism. Nano-Micro Lett..

[6] Krol A., Pomastowski P., Rafinska K., Railean-Plugaru V., Buszewski B. (2017). Zinc oxide nanoparticles: Synthesis, antiseptic activity and toxicity mechanism. Adv. Colloid Interface Sci..

[7] Kaur M. (2021). Zinc oxide nanoparticles: Green synthesis, characterization and biomedical applications. Mater. Sci. Energy Technol..

[8] Ahmed S. (2016). Green synthesis of silver nanoparticles using Azadirachta indica aqueous leaf extract. J. Radiat. Res. Appl. Sci..

[9] Bekele S.G., Ganta D.D., Endashaw M. (2024). Green synthesis and characterization of zinc oxide nanoparticles using Monoon longifolium leave extract for biological applications. Discov. Chem..

[10] Iravani S. (2011). Green synthesis of metal nanoparticles using plants. Green Chem..

[11] Amrulloh H., Fatiqin A., Simanjuntak W., Afriyani H., Annissa A. (2021). Antioxidant and antibacterial activities of magnesium oxide nanoparticles prepared using aqueous extract of Moringa oleifera bark as green agents. J. Multidiscip. Appl. Nat. Sci..

[12] Alamdari S. (2019). Solvent effect on the phytochemical composition and antioxidant activity of plant extracts: A systematic review. Food Chem..

[13] Bouttier-Figueroa D.C., Cortez-Valadez M., Flores-Acosta M., Robles-Zepeda R.E. (2024). Green Synthesis of Zinc Oxide Nanoparticles Using Plant Extracts and Their Antimicrobial Activity. BioNanoScience.

[14] Khan F., Shariq M., Asif M., Siddiqui M.A., Malan P., Ahmad F. (2022). Green Nanotechnology: Plant-Mediated Nanoparticle Synthesis and Application. Nanomaterials.

[15] Thi T.U.D., Nguyen T.T., Thi Y.D., Thi K.H.T., Phan B.T., Pham K.N. (2020). Green synthesis of ZnO nanoparticles using orange fruit peel extract for antibacterial activities. RSC Adv..

[16] Yadav S. (2020). Green synthesis and characterization of zinc oxide nanoparticles using Tradescantia pallida leaf extract and its antimicrobial activity. Mater. Today Proc..

[17] Butnariu M., Quispe C., Herrera-Bravo J., Fernández-Ochoa Á., Emamzadeh-Yazdi S., Adetunji C.O., Memudu A.E., Otlewska A., Bogdan P., Antolak H. (2022). A Review on *Tradescantia*: Phytochemical Constituents, Biological Activities and Health-Promoting Effects. Front. Biosci..

[18] Cano J. (2018). Phytochemical profile and antioxidant activity of Tradescantia spathacea. Rev. Mex. Cienc. Farm..

[19] Matussin S.N., Tan A.L., Harunsani M.H., Mohammad A., Cho M.H., Khan M.M. (2020). Effect of Ni-doping on properties of the SnO_2_ synthesized using Tradescantia spathacea for photoantioxidant studies. Mater. Chem. Phys..

[20] Muñoz-Echeverri L., Campo-Avendaño D., Hoyos-García M., Obregón-Velázquez M., Muñoz-Vergara J., Giraldo-Correa G. (2021). Green synthesis of ZnO nanoparticles with antibacterial activity for functionalizing cotton textiles. Inf. Técnico.

[21] Gberikon G.M., Adeoti L.I., Aondoackaa A.D. (2015). Effect of ethanol and aqueous solutions as extraction solvents on phytochemical screening and antibacterial activity of fruit and stem bark of Tetrapleura tetrapteraon, *Streptococcus salivarus* and *Streptococcus mutans*. Int. J. Curr. Microbiol. Appl. Sci..

[22] Ramos-Arcos S.A., López-Martínez S., Velázquez-Martínez J.R., Gómez-Aguirre Y.A., Cabañas-García E., Morales-Bautista C.M. (2023). Phytochemicals and Bioactivities of *Tradescantia zebrina* Bosse: A Southern Mexican Species with Medicinal Properties. J. Food Nutr. Res..

[23] Ramírez-Rodríguez S.C., Ortega-Ortiz H., Fortis-Hernández M., Nava-Santos J.M., Orozco-Vidal J.A., Preciado-Rangel P.P. (2021). Chitosan nanoparticles improve the nutraceutical quality of triticale sprouts. Rev. Mex. Cienc. Agrícolas.

[24] Brand-Williams W., Cuvelier M.E., Berset C. (1995). Use of a free radical method to evaluate antioxidant activity. LWT-Food Sci. Technol..

[25] Jayachandran A., Aswathy T.R., Nair A.S. (2021). Green synthesis and characterization of zinc oxide nanoparticles using Cayratia pedata leaf extract. Biochem. Biophys. Rep..

[26] Handago D., Zereffa E., Gonfa B. (2019). Effects of *Azadirachta indica* Leaf Extract, Capping Agents, on the Synthesis of Pure and Cu Doped ZnO-Nanoparticles: A Green Approach and Microbial Activity. Open Chem..

[27] Bhuyan T., Mishra K., Khanuja M., Prasad R., Varma A. (2015). Biosynthesis of zinc oxide nanoparticles from *Azadirachta indica* for antibacterial and photocatalytic applications. Mater. Sci. Semicond. Process..

[28] Khan A.U.H., Liu Y., Naidu R., Fang C., Dharmarajan R., Shon H. (2021). Interactions between zinc oxide nanoparticles and hexabromocyclododecane in simulated Waters. Environ. Technol. Innov..

[29] Malik G., Mitra J. (2021). Zinc oxide nanoparticle synthesis, characterization, and their effect on mechanical, barrier, and optical properties of HPMC-based edible film. Food Bioprocess Technol..

[30] Gómez-Garzón M. (2018). Nanomateriales, nanopartículas y síntesis verde. Rev. Repert. Med. Cir..

[31] Tan J.B.L., Yap W.J., Tan S.Y., Lim Y.Y., Lee S.M. (2016). Antioxidant content, antioxidant activity, and antibacterial activity of five plants from the *Commelinaceae* family. Antioxidants.

[32] Rosales-Reyes T., de la Garza M., Arias-Castro C., Rodríguez-Mendiola M., Fattel-Fazenda S., Arce-Popoca E. (2008). Aqueous crude extract of *Rhoeo discolor*, a Mexican medicinal plant, decreases the formation of liver preneoplastic foci in rats. J. Ethnopharmacol..

[33] Sánchez A.M., Carmona M., Prodanov M., Alonso G.L. (2008). Effect of centrifugal ultrafiltration on the composition of aqueous extracts of saffron spice (*Crocus sativus* L.). J. Agric. Food Chem..

[34] Jayakumar S., Mahendiran D., Viswanathan V., Velmurugan D., Rahiman A.K. (2017). Heteroscorpionate-based heteroleptic copper(II) complexes: Antioxidant, molecular docking and in vitro cytotoxicity studies. Appl. Organomet. Chem..

[35] Singh V., Lehri A., Singh N. (2019). Assessment and comparison of phytoremediation potential of selected plant species against endosulfan. Int. J. Environ. Sci. Technol..

[36] Ankamwar B., Kirtiwar S., Shukla A.C., Patra J., Shukla A., Das G. (2020). Plant-mediated green synthesis of nanoparticles. Advances in Pharmaceutical Biotechnology.

[37] Ghamsari M.S., Alamdari S., Han W., Park H. (2016). Impact of nanostructured thin ZnO film in ultraviolet protection. Int. J. Nanomed..

[38] Muthu K., Priya S. (2017). Green synthesis, characterization and catalytic activity of silver nanoparticles using Cassia auriculata flower extract separated fraction. Spectrochim. Acta Part A Mol. Biomol. Spectrosc..

[39] Singh J., Dutta T., Kim K.H. (2018). ‘Green’ synthesis of metals and their oxide nanoparticles: Applications for environmental remediation. J. Nanobiotechnol..

[40] Mukunthan K.S., Balaji S. (2012). Cashew apple juice (*Anacardium occidentale* L.) speeds up the synthesis of silver nanoparticles. Int. J. Green Nanotechnol..

[41] Love A.J., Makarov V.V., Sinitsyna O.V., Shaw J., Yaminsky I.V., Kalinina N.O. (2015). Genetically modified tobacco mosaic virus that can produce gold nanoparticles from a metal salt precursor. Front. Plant Sci..

[42] El-Beltagi H.S., Ragab M., Osman A., El-Masry R.A., Alwutayd K.M., Althagafi H. (2024). Biosynthesis of zinc oxide nanoparticles via neem extract and their anticancer and antibacterial activities. PeerJ.

[43] Islam M.F., Islam S., Miah M.A.S., Huq A.K.O., Saha A.K., Mou Z.J. (2024). Green synthesis of zinc oxide nanoparticles using *Allium cepa* L. waste peel extracts and its antioxidant and antibacterial activities. Heliyon.

[44] Yang X., Cao X., Chen C., Liao L., Yuan S., Huang S. (2023). Green synthesis of zinc oxide nanoparticles using aqueous extracts of *Hibiscus cannabinus* L.: Wastewater purification and antibacterial activity. Separations.

[45] Abdelbaky A.S., El-Mageed T.A., Babalghith A.O., Selim S., Mohamed A.M.H.A. (2022). Green synthesis and characterization of ZnO nanoparticles using *Pelargonium odoratissimum* (L.) aqueous leaf extract and their antioxidant, antibacterial, and anti-inflammatory activities. Antioxidants.

[46] Bindu P., Thomas S. (2014). Estimation of lattice strain in ZnO nanoparticles: X-ray peak profile analysis. J. Theor. Appl. Phys..

[47] Fardood S.T., Ramazani A., Moradi S., Asiabi P.A. (2017). Green synthesis of zinc oxide nanoparticles using Arabic gum and photocatalytic degradation of direct blue 129 dye under visible light. J. Mater. Sci. Mater. Electron..

[48] Vinayagam R., Selvaraj R., Arivalagan P., Varadavenkatesan T. (2020). Synthesis, characterization, and photocatalytic dye degradation capability of *Calliandra haematocephala*-mediated zinc oxide nanoflowers. J. Photochem. Photobiol. B Biol..

[49] Dulta K., Koşarsoy-Ağçeli G., Chauhan P., Jasrotia R., Chauhan P.K. (2021). A novel approach of synthesis zinc oxide nanoparticles by *Bergenia ciliata* rhizome extract: Antibacterial and anticancer potential. J. Inorg. Organomet. Polym. Mater..

[50] Cross J.O., Opila R.L., Boyd I.W., Kaufmann E.N. (2015). Materials characterization and the evolution of materials. MRS Bull..

[51] Gençyılmaz O., Navruz F.Z., İnce S., Abbas A.A., Salim A.H.S. (2024). Comparative evaluation of zinc oxide nanoparticles (ZnONPs): Photocatalysis, antibacterial, toxicity and genotoxicity. J. Photochem. Photobiol. A Chem..

[52] Sachin J., Singh N., Singh R., Shah K., Pramanik B.-K. (2023). Green synthesis of zinc oxide nanoparticles using lychee peel and its application in anti-bacterial properties and CR dye removal from wastewater. Chemosphere.

[53] Singh K., Singh J., Rawat M. (2016). Green synthesis of zinc oxide nanoparticles using *Punica granatum* leaf extract and its application towards photocatalytic degradation of Coomassie brilliant blue R-250 dye. SN Appl. Sci..

[54] Hwang N.M., Jung J.-S., Lee D.K. (2012). Thermodynamics and Kinetics in the Synthesis of Monodisperse Nanoparticles.

[55] Jesús E.R., Aguilar-Méndez M.A., López-Perea P., Guzmán-Mendoza J., Hernández-Martínez V., Quiroz-Reyes N. (2018). Synthesis of silver nanoparticles using aqueous tejocote extracts as reducing and passivating agent. Ing. Agrícola Biosist..

[56] Alamdari S., Sasani-Ghamsari M., Lee C., Han W., Park H.H., Tafreshi M.J. (2020). Preparation and characterization of zinc oxide nanoparticles using leaf extract of *Sambucus ebulus*. Appl. Sci..

[57] Farooq A., Khan U.A., Ali H., Sathish M., Naqvi S.A.H., Iqbal S. (2022). Green chemistry-based synthesis of zinc oxide nanoparticles using plant derivatives of *Calotropis gigantea* (*Giant milkweed*) and its biological applications against various bacterial and fungal pathogens. Microorganisms.

[58] Wu C., Zhang T., Ji B., Chou Y. (2023). Green synthesis of zinc oxide nanoparticles using *Aloe vera* leaves extract and evaluation of ALE-ZnO/regenerated cellulose films antibacterial, antioxidant properties. Preprint.

[59] Fouda A., Saied E., Eid A.M., Kouadri F., Alemam A.M., Hamza M.F. (2023). Green synthesis of zinc oxide nanoparticles using an aqueous extract of *Punica granatum* for antimicrobial and catalytic activity. J. Funct. Biomater..

[60] Thema F.T., Manikandan E., Dhlamini M.S., Maaza M. (2015). Green synthesis of ZnO nanoparticles via *Agathosma betulina* natural extract. Mater. Lett..

[61] Raj A., Lawrence R. (2018). Green synthesis and characterization of ZnO nanoparticles from leaf extracts of *Rosa indica* and its antibacterial activity. Rasayan J. Chem..

[62] Esparza-Muñóz R.A. (2022). Scanning electron microscopy in materials characterization. Cienc. Front..

[63] Reyes Gasga J. (2020). Brief historical review of electron microscopy in Mexico and the world. Nano World Interdiscip. J. Nanosci. Nanotechnol..

[64] Elumalai K., Velmurugan S. (2015). Green synthesis, characterization and antimicrobial activities of zinc oxide nanoparticles from the leaf extract of *Azadirachta indica* (L.). Appl. Surf. Sci..

[65] Vijayakumar S., Vinoj G., Malaikozhundan B., Shanthi S., Vaseeharan B. (2015). *Plectranthus amboinicus* leaf extract mediated synthesis of zinc oxide nanoparticles and its control of methicillin-resistant *Staphylococcus aureus* biofilm and blood-sucking mosquito larvae. Spectrochim. Acta A Mol. Biomol. Spectrosc..

[66] Asmat-Campos D.A. Green synthesis of ZnO nanoparticles and their photocatalytic evaluation of methyl yellow degradability using a low-power UV-A lamp. Proceedings of the 19th LACCEI International Multi-Conference for Engineering, Education, and Technology.

[67] Vanlalveni C., Lallianrawna S., Biswas A., Selvaraj M., Changmai B., Rokhum S.L. (2021). Green synthesis of silver nanoparticles using plant extracts and their antimicrobial activities: A review of recent literature. RSC Adv..

[68] Kołodziejczak-Radzimska A., Jesionowski T. (2014). Zinc oxide—From synthesis to application: A review. Materials.

[69] Sabir S., Arshad M., Chaudhari S.K. (2014). Zinc oxide nanoparticles for revolutionizing agriculture: Synthesis and applications. Sci. World J..

[70] Gnanasangeetha D., Thambavani D.S. (2013). Biogenic production of zinc oxide nanoparticles using *Acalypha indica*. J. Chem. Biol. Phys. Sci..

[71] Cuadros-Moreno A., Pimentel R.G.C., Martínez E.S.M., Fernández J.Y. (2014). Dynamic light scattering in the sizing of polymeric nanoparticles. Lat. Am. J. Phys. Educ..

[72] Rasli N.I., Basri H., Harun Z. (2020). Zinc oxide from *Aloe vera* extract: Two-level factorial screening of biosynthesis parameters. Heliyon.

[73] Yoval L.S., Palacios L.M., Soberanis M.P., Guzmán L.O.S. (2000). Zeta Potential as a Tool for Determining Particle Agglomeration in Sludge Volume Reduction. http://elaguapotable.com/POTENCIAL%20ZETA%20COMO%20UNA%20HERRAMIENTA%20PARA%20DETERMINAR%20LA.pdf.

[74] Srivastav A.K., Kumar M., Ansari N.G., Jain A.K., Shankar J., Arjaria N. (2016). A comprehensive toxicity study of zinc oxide nanoparticles versus their bulk in Wistar rats. Hum. Exp. Toxicol..

[75] Bandeira M., Giovanela M., Roesch-Ely M., Devine D.M., da Silva Crespo J. (2020). Green synthesis of zinc oxide nanoparticles: A review of the synthesis methodology and mechanism of formation. Sustain. Chem. Pharm..

[76] Rajendran R., Mani G., Dhandapani M., Maruthamuthu S., Kalaiselvam S. (2022). Phytochemical profiling and green synthesis of zinc oxide nanoparticles using Azadirachta indica: Optimization, characterization and antimicrobial activity. J. Mol. Struct..

